# Supplementary figures and images for: Small-cell carcinoma in the head and neck region: A propensity score-matched analysis of the effect of surgery
Source: PLoS One. 2024 Oct 24;19(10):e0312455. doi: 10.1371/journal.pone.0312455 (PMC11500905; doi:10.1371/journal.pone.0312455)

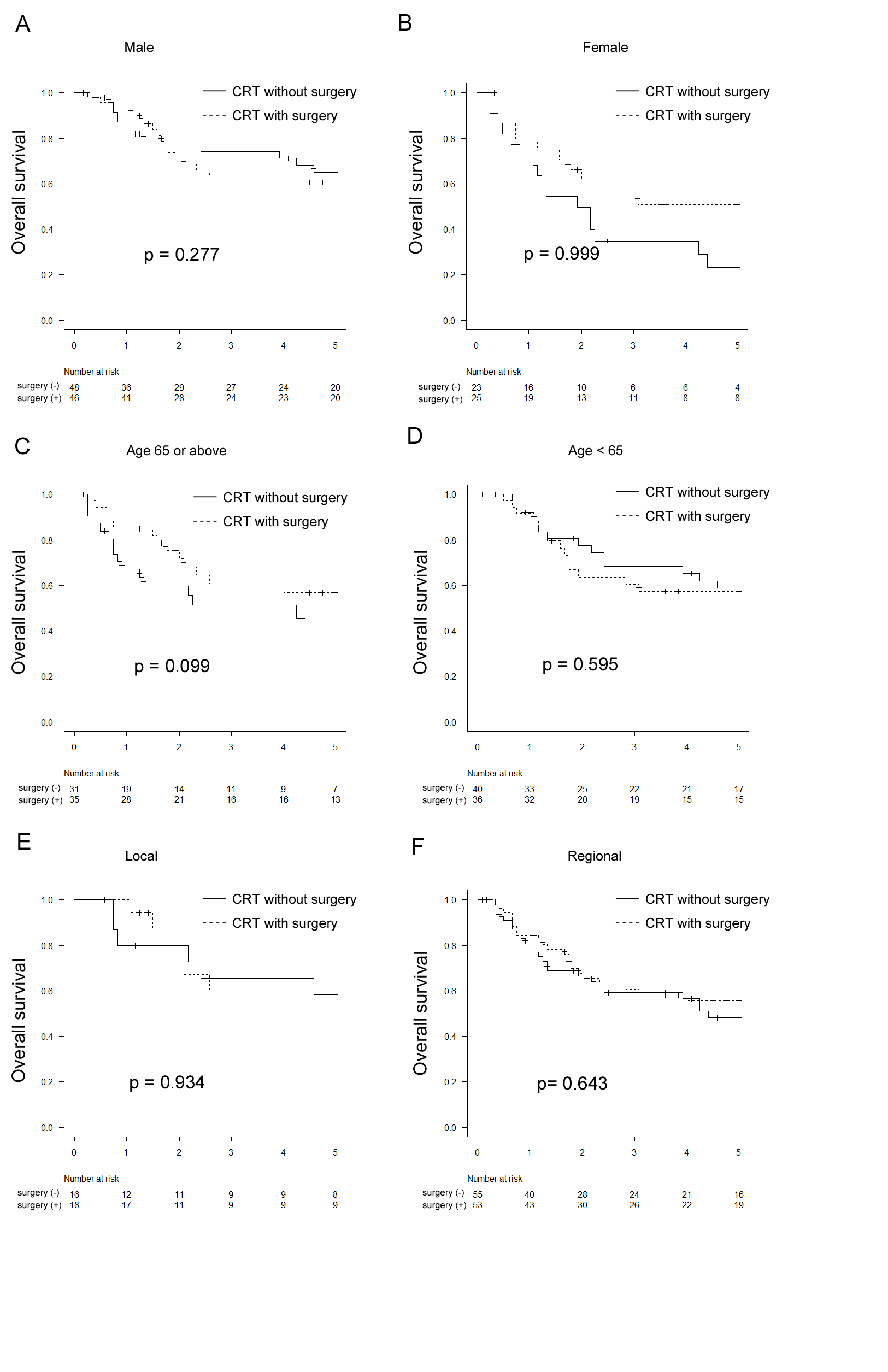

Supplement: S1 Fig — (A) male, (B) female, (C) age ≥ 65 years, (D) age < 65 years, (E) localized disease, (F) regional disease. (TIF) [file pone.0312455.s001.tif]
