# Supplementary material for: Small-cell carcinoma in the head and neck region: A propensity score-matched analysis of the effect of surgery
Source: PLoS One. 2024 Oct 24;19(10):e0312455. doi: 10.1371/journal.pone.0312455 (PMC11500905; doi:10.1371/journal.pone.0312455)
Supplement: Attachment — Submitted filename: reply to reviewer.docx [file pone.0312455.s004.docx]

**We would like to express our sincere gratitude for taking the time to review our manuscript.**

**Reviewer #1:** This manuscript primarily discusses the benefits of surgery for head and neck small cell carcinoma compared to chemoradiation therapy. The use of propensity score matching to adjust for age-related effects in the surgery group is a strong aspect that refines the results. However, there are a few limitations that need to be addressed:

**Response:**

**We thank reviewer 1 for reviewing our manuscript.**

1. Terminology Consistency: Please review lines 57-58 to ensure consistent use of terminology. Specifically, please confirm whether the T, N, M stratification should be referred to as T, N, M stages in accordance with the reference publication by Yu et al.

**Response:**

**In accordance with the reviewer’s suggestion, we revised the manuscript.**

2. Patient Enrollment: Please include a flow diagram to illustrate the patient selection process.

**Response:**

**In accordance with the reviewer’s suggestion, we added a flow diagram.**

3. Propensity Score Matching: The manuscript mentions the use of 1:1 nearest matching to adjust for discrepancies in age and primary sites. However, it should be noted that nearly 40% of the sample data were not matched, indicating the current matching did not fully account for primary sites. So please consider adjusting the parameters in the propensity score matching to make better use of the data. For instance, switching to partial exact matching for primary sites, using Mahalanobis distance in MatchIt, etc. Additionally, there is no evaluation provided for the matched pairs. Please include assessment metrics, such as the distribution of propensity scores before and after matching, to demonstrate the effectiveness of the matching process.

**Response:**

**We acknowledge the reviewer’s concern regarding the 40% data loss with 1:1 nearest neighbor matching. While this approach has limitations, it has been widely used and accepted in prior studies. To address the potential impact of data loss and assess the robustness of our findings, we conducted an additional analysis using Inverse Probability of Treatment Weighting (IPTW), which allowed us to fully utilize the data. The results from the IPTW analysis were consistent with those obtained through nearest neighbor matching**

**Also, we added the kernel plot of the propensity scores (revised figure 2).**

4. Subgroup Analysis: It appears that sex and cancer stages may influence the effect of surgery, as indicated by the univariate factors and in the discussion section. Please consider conducting Kaplan-Meier (KM) curves for these subgroups to verify these effects.

**Response:**

**In accordance with the reviewer’s suggestion, we added the results for survival analyses stratified by clinical variables (supplementary figure 1).**

**Reviewer #2:** In this study, SEER data were used to investigate overall survival and disease-specific survival in patients with locoregional small cell carcinoma of the head and neck treated with chemoradiotherapy (CRT) or CRT plus surgery. Although the study targeted a rare histological type, a sufficient number of propensity score-matched cases (71 in each group) were used for comparison.

It seems that in many cases of CRT plus surgery, small cell carcinoma was diagnosed through surgery and then CRT was added. For clinical decision-making, the prognosis of cases treated with surgery alone and surgery plus chemotherapy is also important. However, when there are multiple divergent treatments, it is difficult to compare them.　 Although this study has the limitation that it was not prospective, it clearly demonstrated that there is no benefit to performing surgery before CRT in locoregional tumors diagnosed as small cell carcinoma by biopsy.
　
This study will be useful to many clinicians and is worthy of publication in the journal.

**Response:**

**We thank the reviewer 2 for reviewing our manuscript.**
